# Comparative transcriptomics of elasmobranchs and teleosts highlight important processes in adaptive immunity and regional endothermy

**DOI:** 10.1186/s12864-016-3411-x

**Published:** 2017-01-30

**Authors:** Nicholas J. Marra, Vincent P. Richards, Angela Early, Steve M. Bogdanowicz, Paulina D. Pavinski Bitar, Michael J. Stanhope, Mahmood S. Shivji

**Affiliations:** 1 Department of Population Medicine and Diagnostic Sciences, College of Veterinary Medicine, Cornell University, Ithaca, NY 14853 USA; 2 Save Our Seas Shark Research Center and Guy Harvey Research Institute, Nova Southeastern University, 8000 North Ocean Drive, Dania Beach, FL 33004 USA; 3 Department of Biological Sciences, Clemson University, Clemson, SC 29634 USA; 4 Department of Ecology and Evolutionary Biology, Cornell University, Ithaca, NY 14853 USA

**Keywords:** Regional endothermy, Adaptive immunity, Gene ontology, Positive selection, RNA-seq, Elasmobranchs

## Abstract

**Background:**

Comparative genomic and/or transcriptomic analyses involving elasmobranchs remain limited, with genome level comparisons of the elasmobranch immune system to that of higher vertebrates, non-existent. This paper reports a comparative RNA-seq analysis of heart tissue from seven species, including four elasmobranchs and three teleosts, focusing on immunity, but concomitantly seeking to identify genetic similarities shared by the two lamnid sharks and the single billfish in our study, which could be linked to convergent evolution of regional endothermy.

**Results:**

Across seven species, we identified an average of 10,877 Swiss-Prot annotated genes from an average of 32,474 open reading frames within each species’ heart transcriptome. About half of these genes were shared between all species while the remainder included functional differences between our groups of interest (elasmobranch vs. teleost and endotherms vs. ectotherms) as revealed by Gene Ontology (GO) and selection analyses. A repeatedly represented functional category, in both the uniquely expressed elasmobranch genes (total of 259) and the elasmobranch GO enrichment results, involved antibody-mediated immunity, either in the recruitment of immune cells (Fc receptors) or in antigen presentation, including such terms as “antigen processing and presentation of exogenous peptide antigen via MHC class II”, and such genes as MHC class II, *HLA-DPB1*. Molecular adaptation analyses identified three genes in elasmobranchs with a history of positive selection, including legumain (*LGMN*), a gene with roles in both innate and adaptive immunity including producing antigens for presentation by MHC class II. Comparisons between the endothermic and ectothermic species revealed an enrichment of GO terms associated with cardiac muscle contraction in endotherms, with 19 genes expressed solely in endotherms, several of which have significant roles in lipid and fat metabolism.

**Conclusions:**

This collective comparative evidence provides the first multi-taxa transcriptomic-based perspective on differences between elasmobranchs and teleosts, and suggests various unique features associated with the adaptive immune system of elasmobranchs, pointing in particular to the potential importance of MHC Class II. This in turn suggests that expanded comparative work involving additional tissues, as well as genome sequencing of multiple elasmobranch species would be productive in elucidating the regulatory and genome architectural hallmarks of elasmobranchs.

**Electronic supplementary material:**

The online version of this article (doi:10.1186/s12864-016-3411-x) contains supplementary material, which is available to authorized users.

## Background

The class Chondrichthyes includes all of the cartilaginous fishes: the chimaeras, sharks, skates, and rays. The extant members of the class comprise at least 1,207 species [1], divided into two major groups: Subclasses Holocephali (chimaeras) and Elasmobranchii (sharks, skates, and rays). Recent molecular dating analyses suggest a split between holocephalans and elasmobranchs at about 420 Mya [2, 3]. Chondrichthyans as a whole, are thought to have diverged from bony vertebrates (Osteichthyes: ray-finned fishes, coelacanths, lungfishes, and tetrapods) approximately 450–475 Mya [2–4]; see however Giles et al. 2015 [5], for evidence of a chondrichthyan/osteichthyan ancestry of 415 Mya. Because of their phylogenetic position in vertebrate evolution, chondrichthyans provide an important reference for our understanding of vertebrate genome evolution. In addition to their ancient history and fundamental position in vertebrate systematics, chondrichthyans possess a wide variety of biological characteristics of note. Such traits include, but are not limited to, the presence of a primitive adaptive immune system, efficient wound healing, and the evolution of regional endothermy in several species.

One of the most rapidly expanding areas of research in elasmobranch biology is in understanding the function of the immune system [6]. Cartilaginous fishes are the most ancient group of vertebrates that possesses an adaptive immune system based on the same B and T cell receptor genes that form the foundation of adaptive immunity in higher vertebrates [7]. However, adaptive immunity in chondrichthyans differs from higher vertebrates (including teleost fishes) in the lack of bone marrow (where B cells typically develop), in the types of immunoglobulins (Ig hereinafter), and in the genomic organization of the underlying genes [8–11]. Additionally, elasmobranchs contain a novel immunoglobulin, referred to as new antigen receptor (or IgNAR), which differs from traditional immunoglobulins in that it lacks light chain molecules and is comprised entirely of heavy chain domains [12, 13]. IgNARs have received considerable interest recently, in regard to their unique structure and the possibility of adapting these molecules for future diagnostic work or drug delivery systems [14–16]. Despite this interest, transcriptomic analyses of the similarities and differences between the elasmobranch immunome and that of higher vertebrates are not currently available.

Regional (or partial) endothermy arose independently in each of the billfishes and tunas (both highly migratory, large bodied teleosts), and the highly migratory, large-bodied lamnid sharks. All three groups have convergently evolved adaptations for increased aerobic capacity, continuous swimming, elevated cruising speed, and heat production and/or retention [17–21]. Although the heart is at ambient temperature in regionally endothermic species, its function is critical to endothermic physiology because of its role in modulating blood flow and oxygen delivery to heat producing tissues (i.e. red muscle) and through the vasculature responsible for the counter-current heat exchange [18]. However, to date the genetic loci that might be associated with this remarkable example of convergent evolution in fishes remain obscure and there are few studies that specifically attempt to investigate this. A recent study of the cytochrome oxidase C subunit (COX) genes found no evidence of molecular convergence across endothermic fishes in these mitochondrial loci involved in aerobic metabolism [22]. Another recent study has shown differences in expression of genes involved in calcium storage and cycling (*serca2* and *ryr2*) in heart tissue of tuna species with different temperature tolerance, with the greatest expression in Pacific bluefin tuna (*Thunnus orientalis*), the species with the greatest cold tolerance of the three tested [23]. In a comparative study of gene expression in heart tissue of Pacific bluefin tuna that were acclimated to cold and warm temperatures, Jayasundara and colleagues found upregulation of genes associated with protein turnover, lipid and carbohydrate metabolism, heat shock proteins, and genes involved in protection against oxidative stress in cold acclimated individuals [24]. This study also detected elevated levels of the *SERCA* enzyme in cold acclimated individuals [24]. Collectively, this suggests the importance of regulating genes involved in metabolism, control of heart contraction and function, and cellular protection against oxidative stress in heart tissue of an organism with an endothermic physiology. Our goal here was to use the heart transcriptome to examine a large repertoire of genes for possible evidence of convergent evolution in regional endothermy, in terms of either genes expressed, or shared genes with a history of molecular adaptation.

Comparative genomics of chondrichthyans remains limited, with a single genome sequence available for the holocephalan, *Callorhinchus milii* [25, 26], and a few additional genome projects in progress (reviewed in [27], including the whale shark *Rhincodon typus* (http://whaleshark.georgiaaquarium.org), white shark, *Carcharodon carcharias* (our laboratory), catshark, *Scyliorhinus canicula* (Genoscope: http://www.genoscope.cns.fr), and the batoid, the little skate, *Leucoraja erinacea* [28]. There are a larger number of transcriptomic and RNA-seq studies, however, these genetic resources are still limited compared to those of other vertebrate taxa [27]. Transcriptome sequence examples include a heart transcriptome of the white shark [29]; brain, liver, pancreas, and embryo from the small-spotted catshark, *S. canicula*, [30, 31]; an embryo of cloudy catshark, *Scyliorhinus torazame* [32]; whole embryo from the little skate [28]; and spleen and thymus from nurse shark, *Ginglymostoma cirratum* [26] and spleen, thymus, testis, ovary, liver, muscle, kidney, intestine, heart, gills, and brain from elephant shark (a holocephalan), *C. milii* [26]. In addition, EST (expressed sequence tag) sequences exist for cell lines derived from *L. erinacea* and the spiny dogfish, *Squalus acanthias* [33].

Interspecific transcriptomic comparisons of many taxonomic groups, and in particular groups with limited genetic resources such as elasmobranchs, are confounded by both the haphazard sampling of different tissues associated with different studies as well as the different technologies used to obtain the sequence data. At present limited comparative data sets of the same tissue type and technology are available across many taxa, however, this is beginning to change and there exist a few important exceptions; see for example, [34–36].

To examine transcriptomic differences between elasmobranchs vs. teleosts and endothermic vs. ectothermic (i.e. non-endothermic) species, we sampled heart tissue since it is a metabolically active tissue, and expression of major components in innate and adaptive immunity have been demonstrated in heart and associated blood tissues [37, 38]. Compared to ectothermic fishes, regionally endothermic fishes such as tunas tend to have an elevated heart rate and this in part supports the maintenance of elevated temperature in some tissues [18, 39]. We hypothesize, therefore, that there are differences in expressed gene content of heart tissue of endothermic species relative to ectothermic species, to compensate for this increased heart rate. Our study included the following seven species: elasmobranchs - white shark (*Carcharodon carcharias*), shortfin mako (*Isurus oxyrinchus*; hereinafter termed mako), great hammerhead (*Sphyrna mokarran*; hereinafter termed hammerhead), and yellow stingray (*Urobatis jamaicensis*); teleosts - swordfish (*Xiphias gladius*), hogfish (*Lachnolaimus maximus*), and ocean surgeonfish *Acanthurus bahianus*; hereinafter termed surgeonfish). Both white shark and mako (Lamnidae, Lamniformes), like other lamnids, display elevated internal temperatures indicative of regional endothermy [40]; the great hammerhead (Sphyrnidae, Carcharhiniformes) and the yellow stingray (hereinafter referred to as ray) (Urotrygonidae, Myliobatiformes) represent the two ectothermic elasmobranchs included in our study. Molecular phylogenetic studies support rays and skates as the sister group to sharks and suggest that this split was approximately 300 Mya [2, 3, 41]. The swordfish (Xiphiidae, Perciformes) is a representative of a regionally endothermic teleost, while both hogfish (Labridae, Perciformes) and surgeonfish (Acanthuridae, Perciformes) are ectotherms.

In comparing these seven heart transcriptomes we had several specific aims. First, we sought to identify differences in expressed gene content that are a reflection of evolutionary taxonomy (e.g. elasmobranchs vs. teleosts). Secondly, we aimed to identify whether there were significant differences involving the comparative groups– i.e., elasmobranchs vs. teleosts and endotherms vs. ectotherms—in the types of genes (as identified by differences in Gene Ontology, or GO) that are expressed, especially in regards to particular phenomena of interest (e.g. adaptive immunity and wound healing in elasmobranchs, metabolic function in endotherms). Finally, we sought to identify genes with a history of molecular adaptation in elasmobranchs and the endothermic taxa in our data set, through the identification of genes under positive selection in the respective lineages.

## Methods

### Tissue and RNA

The shark and swordfish hearts were opportunistically obtained from freshly deceased animals captured by recreational or commercial fishermen independent of our study. Dissection was followed by immediate cold storage in order to limit RNA degradation (see below). The ray heart was opportunistically obtained from independent researchers conducting a study on its reproductive organs. Hearts from the hogfish and surgeonfish were similarly obtained from independent researchers conducting a study on age and growth, and stored in RNA*later®* (ThermoFisher). Heart tissue from all other species was stored at −80 °C. No ethical approval or permit for animal experimentation was required, as the individuals were not sacrificed specifically for this study. At Cornell University, total RNA was extracted from the frozen heart tissue for each species using the Agencourt® RNAdvance™ Tissue Kit. Extractions were conducted according to manufacturer instructions. Briefly, as part of the extraction protocol tissue was homogenized and digested in lysis buffer containing proteinase K. RNA from this digested tissue was bound to paramagnetic beads to remove contaminants prior to treatment with DNase I and subsequent elution of the extracted RNA in nuclease free water. Due to the collection of some samples from fishermen and uncertainty regarding the time since death, we checked for RNA degradation using an Agilent 2100 BioAnalyzer or AATI Fragment Analyzer™ and quantified extractions using a Qubit™ spectrofluorometer. Prior to further processing these extractions were shown to pass internal quality standards for the Agilent BioAnalyzer and AATI Fragment Analyzer and had limited evidence of degradation. The total RNA extracted from each species was then used to prepare Illumina TruSeq RNA sequencing libraries according to manufacturer’s instructions at the genomics facility in the Cornell Biotechnology Resource Center.

### Sequencing and assembly

Two lanes of 2x100 bp paired-end sequencing were conducted on an Illumina Hi-Seq 2500 by the Genomics facility in the Biotechnology Resource Center at Cornell University. Four species were pooled per lane (including an eighth species whose library yielded poor sequencing data and was excluded from further analysis). Following sequencing, reads were separated by species and the program FastqMcf within ea-utils [42] was used to remove sequencing adaptors, trim poor quality bases, and remove poor quality reads using a minimum Phred quality score of 30, minimum trimmed length of 50 bp, and removing duplicate reads with 35 or more identical bases. Each species read pool was then used to assemble a species-specific heart transcriptome using Trinity (default parameters, version r2013-02-25 [43]). Following transcriptome assembly, the program TransDecoder (within the Trinity package) was used to extract the longest likely open reading frame (ORF) for each Trinity transcript using default parameters.

### Transcriptome assessment and annotation

To get an estimate of the completeness of each transcriptome we analyzed each assembly with the tool CEGMA (under default parameters) to assess the presence of 248 Core Eukaryotic Genes (CEGs) [44, 45]. Subsequently, initial annotation for each transcriptome was done with a BLASTP [46] search (e-value ≤1e-06, minimum match length ≥ 33 amino acids) against the Swiss-Prot database. Blast hits were imported into Blast2GO version 3.1 [47, 48], which was used to assign Gene Ontology (GO) terms to transcripts for each species. Following this BLASTP search, we removed all duplicate sequences within each species that shared the same BLASTP hit in the Swiss-Prot database, retaining the longest sequence with the greatest sequence similarity to the reference sequence. This was done to remove sequences that arose from possible assembly errors and to restrict our analyses to gene level comparisons, rather than also include comparisons across putative isoforms. We refer to this as our most conservative data set and these annotations were used for all analyses unless otherwise indicated. For most of the species concerned here it was not possible to collect RNA-seq data from multiple individuals, which precluded the confirmation of true isoform sequences from assembly errors, by looking for cases of shared intraspecific isoform expression.

### Comparison of expressed gene content

To assess expressed gene content shared among species we conducted a clustering analysis to identify sequence clusters between all seven species and an additional chondrichthyan, the elephant shark, *Callorhinchus milii*. This species is a member of the Holocephali, a separate suborder of the Chondrichthyes; we obtained heart RNA-seq data for this species from a recently updated genome assembly of this organism [26]. We subjected the *C. milii* read data to the same FastqMcf trimming and Trinity assembly methods as the RNA-seq data generated in this study. For the clustering analysis we conducted an all against all BLASTP (e-value ≤ 1e-05) search of all protein sequences from the eight species. The results from these BLASTP searches were used in MCLBlastLINE [49], as implemented in [29, 50], to identify homologous sequences between species using an MCL algorithm to cluster protein sequences with an inflation parameter of 1.8 and all other parameters set to defaults. It is possible, using this approach, for paralogues and orthologues to be grouped together in the same sequence cluster; species were considered to share sequence clusters if one or more transcripts from each species were grouped together in the same cluster (hereinafter referred to as an MCL cluster). We tallied all pairwise MCL clusters between all species and those shared between groups of interest (teleost vs. elasmobranch and regional endotherms vs. ectotherms).

In addition to this clustering approach, we sought to identify the enrichment of particular GO terms in elasmobranchs vs. teleosts or involving the regional endothermic species vs. ectotherms. Towards this end, we conducted Fisher’s exact tests through FatiGO (filtering mode set to FDR adjusted *p-*value) [51] within BLAST2GO version 3.1 [47, 48] to test whether particular GO terms were overrepresented in comparisons of the four elasmobranch to the three teleost species or in comparisons of the three regional endotherms to the four ectotherm species. Each test was two-tailed and allowed us to assess which terms were either overrepresented or underrepresented in our focal group, and filtering at *p <* .05 after FDR correction. Using BLAST2GO, these results were also filtered to obtain a list of the most specific GO terms that were significantly enriched. In this filtering step, if there are multiple GO terms in the same GO hierarchy and they are significantly enriched, then only the most specific term will be retained. For example, the term ‘ion channel complex’ would be a more specific term than ‘transmembrane transporter complex’ and ‘transmembrane transporter complex’ would be a more specific term than ‘cellular component’. If ‘ion channel complex’ and ‘transmembrane transporter complex’ were both enriched, then the filtering for the most specific terms would remove the term ‘transmembrane transporter complex’ from the list and only show the term ‘ion channel complex’ as enriched.

### Identification of candidate immunity genes

To classify genes involved in immunity, we assembled a master list of candidate genes involved in both adaptive and innate immune function. This gene list was derived from numerous large-scale mammalian studies that are curated on two databases: InnateDB [52] and the Immunome knowledge base [53]. Using the Swiss-Prot IDs for these candidate genes, we queried our teleost and elasmobranch BLAST data for their presence in the annotations of our most conservative gene set. To ensure that we captured the genes relevant to chondrichthyans, we cross-referenced the adaptive immunity list against the genes identified in the elephant shark genome [26]. Any immunity genes identified in the elephant shark genome that were not in our list were subsequently added to the adaptive immunity list before comparison with our BLAST data.

### Positive selection

Positive selection is the fixation of advantageous mutations driven by natural selection, and is one of the fundamental processes behind adaptive changes in genes and genomes, leading to evolutionary innovations and species differences. We sought to identify cases of positive selection in elasmobranchs and in regional endotherms by conducting the branch sites tests for positive selection on putative orthologues shared between all eight species using the codeml package within PAML version 4.8 [54, 55]. For this analysis, orthologues were defined using the clustering analysis described above, with the additional restriction of only considering clusters with a single sequence from each of the eight species (the seven sequenced here, plus elephant shark). For each cluster we aligned the corresponding coding sequence (cds) using the program Probalign v1.1 [56] with default settings but removing sites where the posterior probability was < 0.6 and retaining only alignments with continuous blocks of aligned sites that covered >50% of the reference protein sequence in the Swiss-Prot database.

Each alignment was then analyzed with two separate models: a null model and an alternative model. For the alternative model, the dn/ds ratio was allowed to vary across the gene and a proportion of sites were allowed to have dn/ds > 1 (model = 2, NSsites = 2, fix omega = 0, initial omega = 1). In the null model, dn/ds was allowed to vary across the gene but fixed at one for the proportion of sites that are allowed to be >1 in the alternative model (model = 2, NSsites = 2, fix omega = 1). We identified selection when the alternative model identified a proportion of sites with a dn/ds >1 and was significantly more likely as determined by a Likelihood Ratio Test (LRT) using the Chi-squared distribution and one degree of freedom. Sites under selection were identified with the Bayes empirical Bayes method (BEB) [57]. Separate runs of each model were conducted, testing for the incidence of positive selection on the branches leading to endothermic taxa (on the branch leading to swordfish and to the lamniformes) and for selection on the branch leading to elasmobranchs. Branch lengths were estimated by PAML for each gene. The program BUSTED [58] was used to confirm the incidence of positive selection via an online server (www.datamonkey.org/busted) using default settings. A multiple sequence alignment is loaded into the BUSTED server, it generates a tree from the alignment, and the user selects foreground branches for assessing positive selection; in each case we selected the branch leading to the elasmobranch ancestor to test for positive selection.

## Results

RNA quality was similar across both wild caught and laboratory collected specimens with little RNA degradation as indicated by high RIN and RQN scores (Average of 7.0 across all libraries and above a minimum of 6.0 for RIN and 5.1 for RQN) on an Agilent 2100 BioAnalyzer or AATI Fragment Analyzer™. The basic sequencing and assembly statistics for the seven heart transcriptomes are summarized in Table 1 and the reads are deposited within the bioproject PRJNA313962. The values reported here follow stringent filtering, with an average of 14,737,476 reads retained per species, and which were subsequently assembled into an average of 121,517 Trinity transcripts per species. To remove non-coding RNA and bioinformatic artifacts from the Trinity assembler we assessed the longest open reading frame (ORF) for each transcript and obtained BLASTP annotations for ORFs from each species, ranging in numbers from 22,491-50,494, with an average of 32,474. The CEGMA analysis (Table 1) indicated that all of our species transcriptomes contained the vast majority of CEGs and these were nearly all “complete” with the possible exception of mako, which still had >90% total coverage but with only 80% of the matches judged as “complete”. A “complete” match represents cases where a transcript has an alignment length ≥ 70% of the CEG protein length.

**Table 1:** Descriptive statistics of quality filtered reads and the subsequent assembly/annotation of 7 heart transcriptomes

Species	Filtered sequence reads	Trinity transcripts	n50	ORFs	Annotated ORFs	Swiss-Prot Proteins	MCL Clusters	Complete coverage of CEGs	Total Coverage of CEGs
White shark	23,288,212	174,288	2,294	46,825	34,986	11,705	7,021	95.2%	98.0%
Great hammerhead	22,977,042	179,367	2,649	49,992	37,700	10,991	6,535	96.4%	98.4%
Mako	10,802,234	101,534	779	30,354	22,491	11,135	6,980	80.2%	90.7%
Ray	12,921,908	104,957	2,004	31,672	23,265	10,651	6,437	98.0%	99.2%
Swordfish	16,524,630	144,990	2,778	63,771	50,494	11,717	6,788	98.0%	100%
Surgeonfish	8,983,274	81,222	2,864	42,302	34,302	9,530	5,999	91.1%	94.4%
Hogfish	7,665,032	64,264	2,168	28,403	24,077	10,412	6,184	87.9%	94.0%

Trinity transcripts refer to the initial number of transcripts in the Trinity assembly, which were then filtered for those containing the longest open reading frames. The translation of these transcripts was then annotated with BLASTP against the Swiss-Prot database and the number of hits to unique Swiss-Prot entries was recorded; if more than one transcript matched the same Swiss-Prot entry then the longest and most significant match was retained. A CEGMA analysis was conducted to evaluate the coverage of Core Eukaryotic Genes with complete coverage representing the proportion of CEGs with “complete” matches and the total coverage representing the percentage of CEGs that had complete or partial matches in the transcriptome

**Fig. 1:**
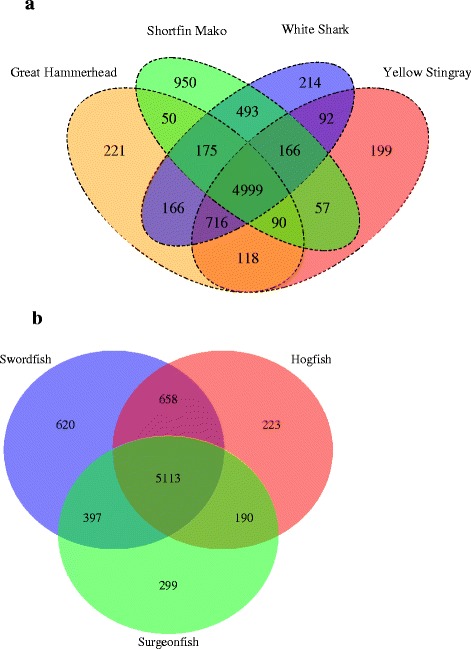
**a** Venn diagram of the MCLBlastLine sequence clusters present in elasmobranchs and how they are distributed among the four elasmobranch species. **b** Venn diagram of the MCLBlastLINE sequence clusters present in teleosts and how they are distributed among the three teleost species

**Fig. 2:**
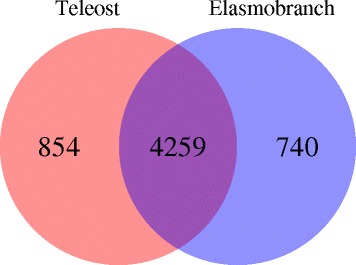
Venn diagram of the MCLBlastLINE sequence clusters shared between teleosts and elasmobranchs (intersection of the diagram) as well as those unique to each of the groups

The MCLBlastLINE analysis resulted in sets of homologous clusters of proteins, which were compared across taxa. Considering first elasmobranchs, more clusters were uniquely shared between mako and white shark relative to white shark and hammerhead (493 and 166 respectively; Fig. 1a). Mako and hammerhead shared many fewer homologues; this combination of similarities and differences of clusters across the shark species included in our dataset is both a reflection of the closer evolutionary relationship of mako and white shark, compared to hammerhead, and of the lower output associated with the mako sequencing run. Mako had the largest number of unique clusters (950) among elasmobranchs (possibly a reflection of the somewhat poorer quality of the data for this species; see for example mako n50, Table 1). Despite rays being separated from sharks by about 300 million years of evolution [2, 3, 41], the yellow stingray had a similar number of unique clusters (199) as white shark (214) and great hammerhead (221). The shared set of elasmobranch heart transcriptome MCL clusters was 4,999 (Fig. 1a), and a similar sized core set was apparent in the comparison involving the three teleosts (5,113, Fig. 1b). A large number of these MCL clusters were shared between all seven species (4,259, Fig. 2) with a similar number unique to teleosts, as well as to elasmobranchs (Fig. 2). The number of clusters restricted to only elasmobranchs and only teleosts, represented 14.8% and 16.7% of the complete elasmobranch and teleost clusters, respectively. When looking at the clusters shared among endothermic species we found 5,192 core clusters shared between the three species (white shark, mako, and swordfish). There were 4,259 clusters shared between endotherms and ectotherms (i.e. all seven taxa) yielding 933 (18% of the total) clusters unique to endotherms and 473 (10% of the total) unique to ectotherms (Additional file 1: Figure S1 and Additional file 2: Figure S2).

### GO content and enrichment tests

**Fig. 3:**
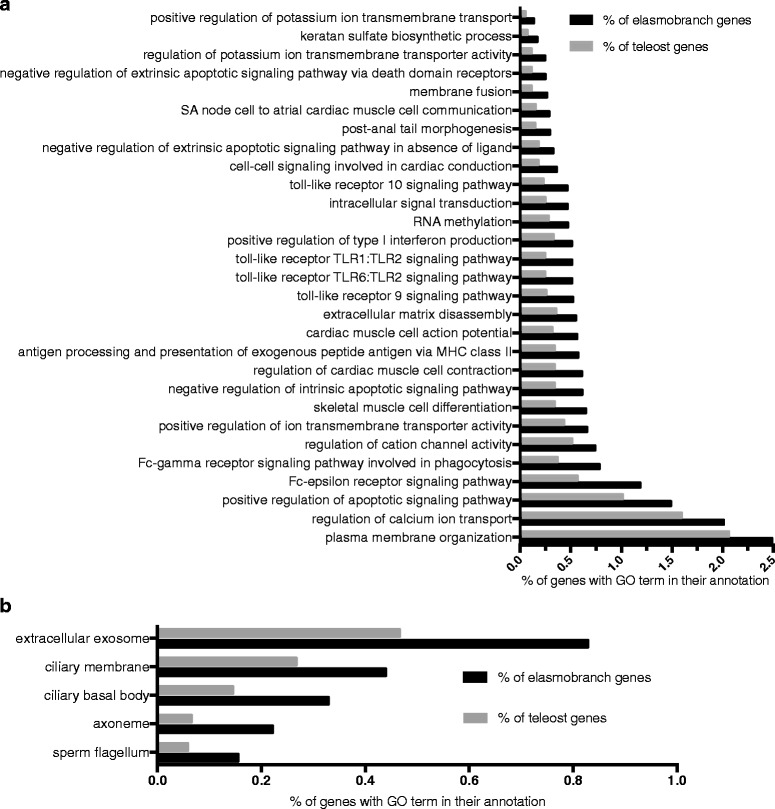
**a** Histogram of the most specific Biological Process GO terms that were found to be significantly enriched in elasmobranchs. Enrichment was judged significant by a Fisher’s exact test and a FDR < .05 after filtering for the most specific terms. **b** Cellular Component GO terms enriched in elasmobranchs after filtering for the most specific terms

**Fig. 4:**
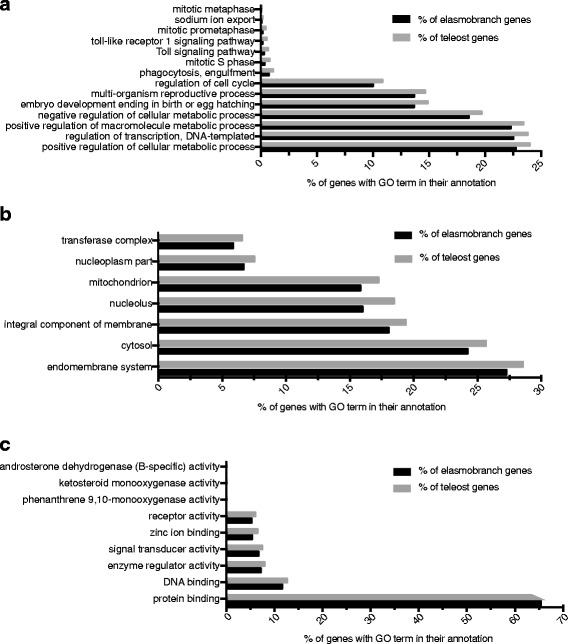
**a** Histogram of the most specific Biological Process GO terms that were found to be significantly enriched in teleosts by a Fisher’s exact test at an FDR < .05 and after filtering for the most specific terms. **b** Cellular Component GO terms enriched in teleosts after filtering for the most specific terms. **c** Molecular Function GO terms enriched in teleosts after filtering for the most specific terms

Additional file 3: Figure S3, Additional file 4: Figure S4, and Additional file 5: Figure S5 show the top 10 most prevalent GO terms for the three main GO categories: Biological Process (BP), Molecular Function (MF), and Cellular Component (CC) in elasmobranchs and teleosts (a. and b. in each fig., respectively). On the whole, teleosts and elasmobranchs share many of the same GO categories and the same proportions of their transcriptomes are annotated with the most prevalent GO terms. However, there does appear to be limited variation within groups (e.g. within elasmobranchs) for the highest frequency GO terms. Enrichment tests did detect statistical differences (i.e. between both elasmobranchs vs. teleosts and endotherms vs. ectotherms) in the representation of GO terms present in lower frequencies within the transcriptomes. A Fisher’s exact test revealed that a total of 93 GO terms were enriched in elasmobranchs (Additional file 6: Table S1) and 97 were enriched in teleosts (Additional file 6: Table S2) after an FDR correction (<.05 post FDR). When filtering these for the most specific GO terms, there were 34 that were enriched in elasmobranchs (four additional terms were removed that were linked to possible symbionts or contaminants). A total of 29 of these terms belonged to the BP category, five were in the CC category; the proportion of genes from elasmobranchs and teleosts that were annotated with these terms is displayed in Figs. 3a and b, respectively. There were 30 GO terms that were enriched in teleosts when filtering for the most specific GO terms (two additional terms were linked to possible symbionts or contaminants), of these 14 were BP terms, seven were CC terms, and nine were MF terms (Figs. 4, a, b, c).

Among the most specific GO terms enriched in teleosts, only two are related to innate immunity (“Toll signaling” and “Toll-like receptor 1 signaling”), and an additional GO is present that may be associated with pathogen removal (“phagocytosis, engulfment”). In contrast, six different GO terms were enriched in elasmobranchs that are involved in innate immunity (five of which are various Toll-like receptor signaling pathways, and the sixth is “positive regulation of type I interferon production”). Additionally, three adaptive immunity GO terms were enriched in elasmobranchs (“Fc-epsilon receptor signaling pathway”, “Fc-gamma receptor signaling pathway involved in phagocytosis”, and “antigen processing and presentation of exogenous peptide via MHC class II”). These terms are all involved in antibody-mediated immunity; either in recruitment of immune cells (Fc receptors) or in antigen presentation.

**Fig. 5:**
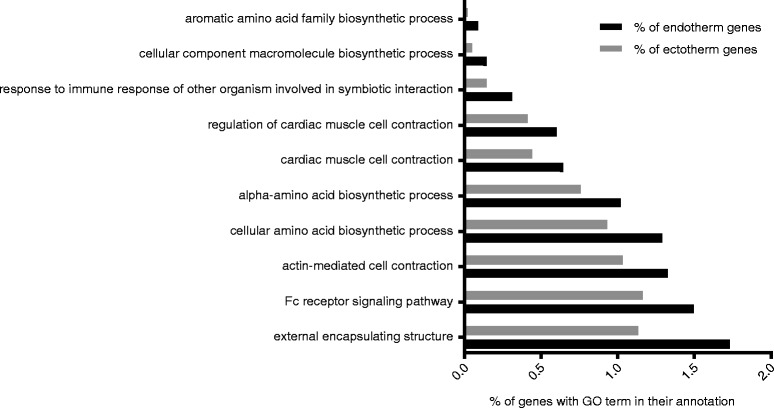
Results of Fisher’s exact test showing the enrichment of GO terms in endotherms. This includes all terms (BP, CC, and MF categories) and at an FDR < .05

The Fisher’s exact test involving the endotherm vs. ectotherm comparison yielded 15 GO terms that were enriched in endotherms (five of which were driven by possible xenobiotics, e.g. bacterial contaminants, pathogens, or commensal organisms, and removed; Fig. 5) and when considering the most specific terms, seven were enriched in endotherms (one CC, six BP); see Additional file 6: Table S3). Although relatively few GO terms were enriched in endotherms, several are of considerable interest including terms describing genes involved in regulation of cardiac muscle cell contraction.

### Unique gene content

In addition to characterizing gene content by clustering analyses and looking at gene ontology information to determine possible functional differences between the transcriptomes, we looked at genes whose expression was restricted to elasmobranchs or to endotherms. We identified 262 Swiss-Prot annotated genes that were restricted to elasmobranchs, three of which were from possible xenobiotics (i.e. sequences that resulted from possible microbial contaminants, pathogens, or symbionts present in the tissue sample). The 259 remaining Swiss-Prot annotated genes that were present in all elasmobranch transcriptomes are listed in Additional file 6: Table S4. These genes span a variety of functions, as indicated by their GO annotations, which included metabolic, gene regulatory, and immunity related terms, among others. There were a few genes (19) that were uniquely expressed in endotherms (listed in Additional file 6: Table S5) with several playing possible roles in energy metabolism.

### Immune genes

**Fig. 6:**
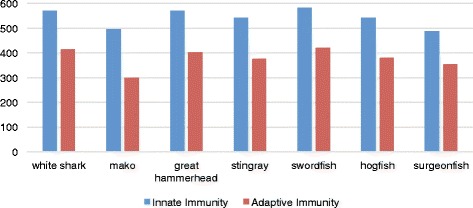
Histogram of the number of candidate immune genes present in each of the species transcriptomes. Candidates were identified as genes having immune functions in model species and listed on InnateDB or the Immunome knowledge base

We also searched for the presence of candidate genes involved in innate and/or adaptive immunity in elasmobranchs and teleosts. In particular, we searched for the presence of 911 innate immunity genes and 862 adaptive immunity genes, with 272 of these being present in both categories. When we cross-referenced our list of candidate genes with the annotations of our most conservative gene lists, we identified 736 innate immunity genes and 599 adaptive immunity genes present in at least one of our seven heart transcriptomes (Fig. 6 shows the distribution of these numbers across all seven species). This included 404 innate immunity genes and 217 adaptive immunity genes present in all four elasmobranch species. Within these there were 17 innate and seven adaptive immunity genes whose expression were absent in heart tissue of teleosts, pointing to a substantial proportion of the heart expressed gene content that is unique to elasmobranchs, being due to expression of immunity related genes (24 of 259 unique elasmobranch genes). In addition to the immunity genes that were expressed in all four elasmobranchs and none of the teleosts; there were 48 additional innate immunity and 37 additional adaptive immunity genes whose expression were absent in teleosts but present in two or more of our elasmobranch species.

### Positive selection

After requiring a single sequence to be present for each species prior to multiple sequence alignment, we were left with 1,332 MCL clusters as possible input for testing positive selection. A further 400 MCL clusters were unable to produce alignments that contained continuous sequence for all eight species, leaving 932 genes that were used as input for Probalign to build consensus quality alignments. Probalign does an additional filtering by removing poor quality alignments, and we further required all alignments to cover > 50% of the sites in the Swiss-Prot reference sequence. After all of these careful filtering steps we were left with 472 high quality alignments to test for the presence of positive selection. All alignments that yielded evidence of positive selection were individually inspected for possible alignment errors and those with obvious misalignments (e.g. inappropriate insertions or deletions) were removed from the dataset.

Analysis of the elasmobranch branch yielded a number of genes that had significant evidence of positive selection (34 genes) with three genes remaining significant after FDR of 0.05, and six at an FDR of 0.10 (Additional file 7: Table S6). This included (at FDR *p-*value < 0.05) a gene of importance to immune function, legumain (*LGMN*, image of alignment available in Additional file 8: Figure S6*)*, which had several sites under selection as identified by BEB. This gene is of particular interest since it was one of our candidate genes in both the innate and adaptive immunity gene lists, and it plays a role in some of the antigen processing steps that were enriched in the elasmobranch GO annotations. The other two genes that displayed strong evidence of positive selection were *Tim22* (mitochondrial import inner membrane translocase subunit TIM22, image of alignment available in Additional file 9: Figure S7) and *Bag1* (BAG family molecular chaperone regulator 1, image of alignment available in Additional file 10: Figure S8), which are involved in transporting proteins into the mitochondria and in regulating various cellular signaling pathways such as apoptosis, respectively.

To provide further confirmatory evidence of positive selection on legumain in elasmobranchs, we employed the program BUSTED [58], looking for episodic positive selection along the branch leading to the elasmobranch ancestor. This test confirmed the branch-site test results, finding significant evidence of diversifying selection (*p =* 0.008) with 11% of sites having a dn/ds of 2.65, indicating an elevated rate of non-synonymous substitutions for our selected branch. The Bayes Empirical Bayes (BEB) analysis conducted during the codeml analysis of legumain indicated that two sites had > 95% probability of being under positive selection in elasmobranchs. These two sites are residues 105 and 261 in the human legumain protein sequence. Neither amino acid is part of the active site (which is composed of a cysteine at residue 189, a histidine at 148, and an asparagine at 42 [59]). However, both sites under selection in elasmobranchs reside at the start or end of a possible helix motif in the secondary structure for the human legumain protein as described on its Swiss-Prot entry. The change at residue 261 is located within the fifth alpha helix of the protein and is also two amino acids before a glycosylation site in the protein [59].

The positive selection analyses on endotherms yielded nine genes where the alternative model was significantly better than the null model and that had a proportion of sites with a dn/ds >1 (Additional file 7: Table S7). One of these genes, catalase (*CAT*), remained marginally significant at a false discovery rate cutoff of 0.10, and is involved in removing oxidative species from the cell, an important complementary process to elevated metabolic function and respiratory rate in an endothermic species [60–63].

## Discussion

Our goal here was to characterize transcriptomic differences between elasmobranchs and teleosts, and more specifically, to identify expressed gene differences related to immune system function between these two taxonomic groups. In addition, by including representative species of both ectotherms and endotherms from each of the two lineages, we aimed to identify expressed loci that might have evolved convergent roles linked to endothermy. In so doing, we conducted transcriptome sequencing and assembly, which provides significant genetic resources for several taxa, and particularly the elasmobranchs, where transcriptomic/genomic resources were previously lacking.

From our assembly statistics it was apparent that the species with the greatest number of sequencing reads yielded many more assembled transcripts, ORFs, and annotated ORFs. However, if we consider the n50 statistics, most species were quite similar with n50 values between two and three kb (with the exception of mako shark at 779 bp), which are on par with similar recent RNA-seq experiments in other non-model species [64] and larger than single tissue RNA-seq assemblies in spotted catshark [31]. The number of unique Swiss-Prot annotations was similar across species (ranging from 9,530 to 11,717, and a mean = 10,877 genes, Table 1) and agrees with similar estimates in heart transcriptome studies in non-model species [65]. We identified 5,358 Swiss-Prot gene annotations that were shared between all seven of our species, indicating that between 46-56% of gene annotations in each species were shared among the rest. When looking at our MCL clusters, the number of clusters that included all species represented from 61-71% of the transcriptome for each species.

In terms of shared orthologues, comparisons to other studies can be difficult to make because large interspecific comparisons are still somewhat nascent, and there are often differences in sequencing technology, output, or ortholog retrieval. Furthermore, the physiological and developmental state from which the animals were sampled will have a significant bearing on the expressed profile of each species [66]. Nonetheless, several studies are worth comparing to our results. Zhang et al. [67] recently compared interspecific spleen transcriptomes of house finch and zebra finch (same order, different families) and found that less than half of all genes expressed in either species were expressed in both species. Similarly, Elmer et al. [68] reported approximately 50% orthologs in transcriptomes of two congener species of cichlid fishes. Thus, the proportion of expressed heart loci that were shared amongst our species is not atypical compared to other studies, especially considering the number of species sampled herein.

We observed that although most species shared a core set of genes (about 50% of gene annotations in each species are shared across the seven species) expressed in heart tissues, there was also a set of genes whose expression was limited to each species and/or group of species. It is possible that differences between species and/or groups could be driven by either (or both) the presence/absence of these hundreds of genes or by regulatory mechanisms driving differential expression [69, 70]. Evaluating whether expression differences between taxa was more of a determining factor than gene presence/absence would require sampling of multiple individuals of each taxon and confirmation of expression values with additional methods such as qPCR. However, and particularly for this sort of tissue, such sampling is not feasible given the difficulties of accessing these large-bodied species in the wild and the conservation status of the sharks, thus we restricted our analysis to the presence/absence rather than levels of expression. Nonetheless, our characterization of the differences in GO term representation, genes whose expression was limited to particular groups, and analysis of positive selection identified genes and pathways that may be of importance to biological differences between these taxa.

### Genetic differences between elasmobranchs and teleosts

The differences in the genes that are expressed solely in elasmobranchs, the enriched GO terms, and the genes under positive selection provide biological insights regarding possible distinguishing factors between elasmobranch and teleost lineages. In particular, a number of immunity related genes differed in their presence and sequence evolution in elasmobranchs. The number of GO terms enriched in elasmobranchs and in teleosts are relatively similar, however, between each of these two sets of genes there are differences that have a direct link to adaptive and innate immunity. It is known that elasmobranchs have a different genomic organization of various adaptive immunity genes compared to other vertebrates [8, 10, 11]. Our GO enrichment results indicate that these genetic differences in adaptive immunity of elasmobranchs exist not only at the genomic but also at the transcriptional level. It is important to identify these differences since it can lead to a better understanding of both the ancestral mechanisms underlying adaptive immunity and those characteristics which have been selectively maintained over an evolutionary timescale that far outstrips that of mammals [6].

The differences in the immunity gene repertoire between teleosts and elasmobranchs was not only apparent in GO category differences but was also evident in the presence of immunity related genes whose expression was restricted to several and/or all elasmobranchs. Similar numbers of candidate adaptive and innate immune genes are present across elasmobranchs and teleosts (Fig. 6). However, when looking at the genes that were expressed only in elasmobranchs, several interesting immunity genes are apparent. This included genes such as “MHC class II antigen, DP beta 1 chain”, ‘Ig gamma 2 chain c region’, and ‘Interferon gamma receptor 1’ which echo the enrichment of GO terms associated with antigen presentation and Fc-receptor signaling in elasmobranchs. This also included genes that were not present in the elephant shark genome. Specifically, five of the seven adaptive immune genes restricted to the four elasmobranchs and 30 of the 37 adaptive immunity genes expressed in two or more of the elasmobranchs (but absent from teleosts), were absent in the reported elephant shark genome. This points to possible differences in immune gene content between elasmobranchs and holocephalans that requires confirmation in a comparison between whole genome sequences of representative species from these two suborders of the Chondrichthyes.

Immunoglobulins in elasmobranchs have received attention in the literature due to differences with higher vertebrates in genomic arrangement and the presence of a novel class of immunoglobulins (new antigen receptor, IgNAR, [7, 8, 10, 11]). We did not detect the presence of IgNAR in our Swiss-Prot annotations; however, this does not preclude the presence of the underlying genes in these species. We did detect three different IgW genes whose expression was limited to one or more elasmobranchs. IgW is similar in form to IgD in mammals but is largely restricted to cartilaginous fish [71–73]. This included a secretory form of the IgW protein that was present in all elasmobranchs, except ray, suggesting its consistent expression. Additionally, there are multiple IgG c-domain genes that have significant matches to sequences in the heart transcriptomes of two or more of our elasmobranch species. Although IgG genes are thought to have arisen in amphibians [74, 75], a form of this molecule has been observed in camels [76] that behaves similarly to IgNAR. Instead of the classical two heavy chain, two light chain, formation of most antibodies, both the camelid IgG and the shark IgNAR are composed of only heavy chains. Given this information and the paucity of elasmobranch IgNAR sequences in Swiss-Prot (only one sequence from nurse shark, *Ginglymostoma cirratum*), we suspect that some of these IgG sequences probably represent Ig molecules typical of cartilaginous fishes, with IgNAR or IgW domains that are not well represented in the curated Swiss-Prot database. Indeed, several of these sequences have better matches to IgNAR or IgW proteins in the NR database. This highlights the need for further identification of all of the requisite domains of IgNAR and the need for additional exploration of these genes in elasmobranchs from a genomic perspective.

Our analyses identified significant evidence of positive selection on several elasmobranch genes including legumain (*LGMN*), which plays an important role in various aspects of immunity, including processing antigens for presentation by MHC class II [59, 77, 78]. Not only is legumain of interest because of its immune functions but it has also been reported to be overexpressed in several cancers [79] and used as a target to eliminate tumor associated macrophages [80]. Elasmobranchs are claimed to have the lowest incidence of malignant neoplasia (tumors) of any vertebrate group [81], however, this remains highly controversial [82], and insufficient information exists from which to base any firm conclusions. At the same time, it is of some note, that there are several publications reporting various shark extracts as having anti-tumorigenic properties [83–85]. Over-expression of legumain occurs in tumor-associated macrophages and is thought to contribute to the tumor promoting inflammation associated with most malignancies [80, 86]. In addition to over or under expression of a gene, altering the structure of the protein, through key substitutions, driven by the action of natural selection, could change its overall function or more subtly alter its role. It is quite possible that the evidence for molecular adaptation that we detect in legumain reflects a modified role in elasmobranchs relative to other vertebrates. We feel it will be important to evaluate the purpose and effect of adaptive changes in this protein in elasmobranchs, providing insight on the impact of these changes on legumain’s role in immunity and its interplay with macrophages. Another gene with a potential role in cancer, which was also under positive selection, and remained significant after FDR correction, was *Bag1* (Bcl2 associated athanogene). *Bcl2* is an oncogene that encodes a membrane protein that blocks a step in a pathway leading to apoptosis [87]. *Bag1* encodes a protein that binds to *Bcl2* and enhances the anti-apoptotic effects of *Bcl2* [88]. Apoptosis, or programmed cell death, is a process that eliminates dysfunctional cells and one of the hallmarks of cancer is the ability of malignant cells to evade apoptosis. One of the proposed modes of action of epigonal (shark lymphomyeloid tissue) cell medium as an anti-tumorigenic substance is in inducing apoptosis [85]. This suggests the possible conjectural hypothesis that positive selection on *Bag1* in elasmobranchs may have altered its tendency to enhance the anti-apoptotic effects of *Bcl2*.

In addition to immunity related functions, annotations of elasmobranch specific genes included terms related to various other roles such as cellular responses (e.g. “signal transduction”), transport (e.g. “intracellular protein transport”), and control of gene expression (e.g. “positive regulation of transcription from RNA polymerase II promoter”). This also included genes not only restricted to elasmobranchs, but genes under positive selection on the elasmobranch branch. Of the three genes that had evidence of positive selection, two of these also lacked an obvious immune function. One of these we already discussed—*Bag1*—the other is *Tim22*, which encodes part of the identically named TIM22 complex. This complex is necessary to transport nuclear encoded proteins that are then embedded in the inner mitochondrial membrane [89]. Differences in the function of this gene could have impacts on basic cellular functions such as cellular respiration and energy production.

It is also possible that some of the differences identified in the elasmobranch transcriptomes, compared to teleosts, are related to their role in wound healing. Sharks, rays, and skates are thought to have the ability to heal wounds relatively quickly, based on various anecdotal reports and observational studies [90–94]. However, the molecular mechanisms of wound healing in elasmobranchs are unclear. In mammals wound repair consists of several distinct stages including an inflammatory response during which various innate and adaptive immune cells such as neutrophils, macrophages, and lymphocytes are recruited to the wound site [95]. The proper timing of these stages is necessary for effective healing, and an important inhibitory factor in the overall process is the development of infection, which can lead to further damage or prolonged inflammation [96]. Thus genes and pathways associated with promoting and controlling inflammation as well as those involved in clearing cellular debris and pathogens are critical to the wound healing process. Some of the processes that we have highlighted as enriched GO terms in elasmobranchs may play an integral complementary role in preventing infection and perhaps even in direct regulation of wound repair. Previously, we discussed the enrichment of several GO terms such as those associated with Fc receptor signaling, and in particular the “Fc-gamma receptor signaling pathway involved in phagocytosis”. Fc-gamma receptors are expressed on a variety of immune cells including neutrophils, mast cells, macrophages, and dendritic cells. When these receptors bind to an antibody/antigen complex they can initiate a variety of responses from stimulating inflammation to initiating phagocytosis of foreign cells (reviewed in [97]), that are important factors in preventing infection and ensuring proper progression of the wound healing process. Thus the genes associated with this process may have roles in wound healing that are complimentary to and/or work in concert with their function in elasmobranch immunity.

Another possible connection between the elasmobranch enrichment results and wound healing is with regard to MHC class II. GO processes associated with antigen presentation by MHC class II were enriched in elasmobranchs, one MHC class II gene had expression restricted to elasmobranch species, and *LGMN* (also involved in processing antigens for presentation by MHC class II) was under selection in elasmobranchs, serving to highlight the importance of the MHC class II complex in elasmobranchs. Interestingly, there is experimental evidence in mouse models showing that wound healing is impaired in the absence of MHC class II. Schäffer et al. [98] demonstrated the importance of MHC class II in the healing of wounds to the skin; they found that wound collagen deposition and wound breaking strength were impaired in MHC-class-II-knockout mice. A study of myocardial infarction in mice found that the absence of CD4^+^ T-cells (which interact with MHC class II) in CD4 knock-outs, resulted in greater mortality and abnormal collagen formation compared to wild-type mice, with even greater mortality in mice that lacked all four MHC class II genes [99]. These studies indicate the importance of MHC class II to not only adaptive immunity but also, more specifically, to tissue repair after injury. To date our understanding of the MHC in elasmobranchs is limited to a few species, and the genomic organization of the region is relatively unknown [100]. Future efforts are needed to understand the organization and function of the MHC in elasmobranchs as part of understanding immune response and the mechanisms by which elasmobranchs are able to regulate efficient healing.

### Gene content associated with regional endothermy

Our hypothesis is that there should be patterns, either in terms of gene content/annotation or in terms of positive selection, that are shared by regionally endothermic fishes, reflecting the convergent evolution of mechanisms that raise the temperature of some organs above ambient. Although the heart is often at ambient temperature in endothermic fishes such as tunas [18, 19, 101], it is still required to supply oxygenated blood to the metabolically active tissues, with accompanying demands placed on its function in regionally endothermic fishes, and with associated impacts on gene expression [24]. Thus we expected that the heart transcriptomes would contain gene content and annotation differences in regional endotherms relative to ectotherms. Indeed, we did identify shared molecular characteristics in the regional endotherms, in terms of GO differences compared to ectotherms, and in terms of genes whose expression were restricted to the endothermic species. There were a total of 15 GO terms enriched in the endothermic taxa involved in our comparison, including a number of categories of likely biological importance to an endothermic physiology, such as the terms “cardiac muscle cell contraction” and “regulation of cardiac muscle cell contraction”. This coincides with expectations that endothermic species have increased cardiac function to accommodate increased metabolism, heart rate, and overall activity [18, 39, 63, 102–104].

There were a total of 19 genes whose expression was restricted to endotherms. The GO terms that describe these genes included several BP terms linked to metabolism, including for instance, the expression of two genes, *ELOVL6* and *TM6SF2*, restricted to endotherms in our dataset and that are involved in lipid metabolism. *ELOVL6* encodes ‘Elongation of very long chain fatty acids protein 6’, which catalyzes the elongation of fatty acids [105]. The function of *TM6SF2* is not well characterized, but it has recently been shown that in humans this gene regulates triglyceride secretion and fat metabolism in the liver [106] and certain variants have been associated with lowered risk of myocardial infarction [107, 108]. In addition to these genes, *MGAM2,* a gene annotated with the GO term: “carbohydrate metabolic process”, was also expressed only in the endotherms. Though there is little information on this gene, its similarity to its paralogue, *MGAM* and its structure have led to its description on Swiss-Prot as being involved in hydrolysis of 1,4-alpha-D-glucose. Considering the higher energy needs and higher metabolic rates of endothermic fishes relative to ectothermic fish [19], the expression of these metabolic genes in the endothermic species may reflect the need for efficient energy storage and metabolism in these species. Elevated expression of genes dealing with lipid and carbohydrate metabolism were also detected in cold acclimated Pacific bluefin tuna, highlighting the importance of metabolic genes to heart function during maintenance of elevated temperature in other tissues of partially endothermic species [24]. The increase in expression of lipid metabolism genes in cold acclimated fish was suggested to lead to an increase in lipid formation and changes in membrane composition [24]. It is possible that some of the genes identified here (e.g. *ELOVL6*), known for their function in other tissues, are playing a role in these metabolic and structural processes in heart tissue of endothermic fishes.

In addition to this evidence suggesting the importance of cardiac function and lipid metabolism in the regional endotherms, we identified limited evidence of positive selection on the gene that encodes the protein catalase. This enzyme is responsible for breaking down hydrogen peroxide and thus preventing damage to the cell from oxidative species [61]. For a species with increased metabolic rate this would be a key process to prevent damage to the cell from H_2_O_2_ produced following electron transport at the end of cellular respiration [60, 63]. The evidence for selection on this gene is, however, marginal after correction for FDR and would require further validation with full-length sequence from lamnid sharks (we possessed only a fragment of the sequence for mako), billfishes, and tunas. Nonetheless, the importance of this gene for protection against oxidative damage makes it an excellent candidate for future study. Indeed, microarray study of the heart transcriptome of the Pacific bluefin tuna at various temperatures have identified changes in the expression of genes dealing with oxidative stress under cold temperatures [24]. This suggests additional study of the evolution of catalase and other genes dealing with reactive oxygen species might well prove a fruitful line of investigation as a secondary adaptation to the metabolic demands in regionally endothermic species.

## Conclusions

In this study we utilized RNA-seq of heart transcriptomes in seven species in order to examine differences in gene content and patterns of positive selection in elasmobranchs relative to teleost fishes, as well as in regional endothermic species. We have uncovered several lines of evidence pointing to differences between elasmobranchs and teleosts in genes underlying notable functional properties. This was particularly highlighted by differences in the expression and evolution of immunity genes in the elasmobranchs, representing the earliest vertebrate lineage with adaptive immunity. Our study also identified genes that may have evolved convergent roles in the phenomenon of regional endothermy, characteristic of a select few elasmobranchs and teleosts. In addition to the biological differences discussed herein, these transcriptome data provide significant genetic resources for future studies in a group (elasmobranchs) that is severely lacking in genomic resources.

## Additional files

Additional file 1: Figure S1a. MCLBlastLINE sequence clusters shared among ectotherms. **Figure S1b.** MCLBlastLINE sequence clusters shared among endotherms. (PDF 573 kb)
Additional file 2: Figure S2. MCLBlastLINE sequence clusters shared between endotherms (left) and ectotherms (right). (PDF 188 kb)
Additional file 3: Figure S3a. Top 10 Biological Process GO terms in elasmobranchs. Bars represent the proportion of contigs in each transcriptome that are annotated with the respective GO term. **Figure S3b.** Top 10 Biological Process GO terms in teleosts. Bars represent the proportion of contigs in each transcriptome that are annotated with the respective GO term. (PDF 311 kb)
Additional file 4: Figure S4a. Top 10 Molecular Function GO terms in elasmobranchs. Bars represent the proportion of contigs in each transcriptome that are annotated with the respective GO term. **Figure S4b.** Top 10 Molecular Function GO terms in teleosts. Bars represent the proportion of contigs in each transcriptome that are annotated with the respective GO term. (PDF 409 kb)
Additional file 5: Figure S5a. Top 10 most abundant Cellular Component GO terms for elasmobranchs. Histograms represent the proportion of contigs in each transcriptome that are annotated with the respective GO term. **Figure S5b.** Top 10 most abundant Cellular Component GO terms for teleosts. Histograms represent the proportion of contigs in each transcriptome that are annotated with the respective GO term. (PDF 290 kb)
Additional file 6: Table S1. List of GO terms that were significantly enriched in elasmobranchs relative to teleosts. The list includes all terms including multiple GO terms that may be enriched from xenobiotics or annotations of the genes function in other tissues. Within the “Category” column, BP is biological process and CC is cellular component. **Table S2.** List of GO terms that were significantly enriched in teleosts relative to elasmobranchs. The list includes all terms including multiple GO terms that may be enriched from xenobiotics or annotations of the genes function in other tissues. Within the “Category” column, BP is biological process, CC is cellular component, and MF is Molecular Function. **Table S3.** List of GO terms that were significantly enriched in endotherms relative to ectotherms. The list includes all terms including multiple GO terms that may be enriched from xenobiotics or annotations of the genes function in other tissues. Within the “Category” column, BP is biological process, CC is cellular component, and MF is Molecular Function. **Table S4.** List of 259 genes that were present in all four elasmobranchs but not present in any of the three teleost species. The Swiss-Prot ID, suggested protein name, and gene name are listed for each gene. **Table S5.** List of 19 genes that were present in all three endotherms but not present in any of the four teleost species. The Swiss-Prot ID, suggested protein name, and gene name are listed for each gene. (XLSX 71 kb)
Additional file 7: Table S6. Genes with evidence of positive selection in elasmobranchs under the branch sites test after FDR filtering that remain significant at *p <* .10. **Table S7.** Genes with evidence of positive selection in endotherms prior to FDR filtering. (DOCX 19 kb)
Additional file 8: Figure S6. Image of the alignment for legumain (*LGMN*) resulting from Probalign, as displayed in Geneious (v. 7.1.7). Bases that are located in gap regions or areas where the alignment is of low confidence are converted to N’s and then removed prior to running the branch sites test. Note that the numbers for residues given in the manuscript correspond to positions in the human reference sequence; the sites under selection according to BEB are flagged with yellow arrows at bases 421 and 889 in the consensus sequence. The alignment file is available upon request. (PDF 4320 kb)
Additional file 9: Figure S7. Image of the alignment for mitochondrial import inner membrane translocase subunit TIM22 (*Tim22*), resulting from Probalign, as displayed in Geneious (v. 7.1.7). Bases that are located in gap regions or areas where the alignment is of low confidence are converted to N’s and then removed prior to running the branch sites test. The sites that are under selection according to BEB are flagged with yellow arrows at bases 139, 184, and 190 in the consensus sequence. The alignment file is available upon request. (PDF 1981 kb)
Additional file 10: Figure S8. Image of the alignment for BAG family molecular chaperone regulator 1 (*Bag1*), resulting from Probalign, as displayed in Geneious (v. 7.1.7). Bases that are located in gap regions or areas where the alignment is of low confidence are converted to N’s and then removed prior to running the branch sites test. The sites that are under selection according to BEB are flagged with yellow arrows at bases 91 and 133 in the consensus sequence. The alignment file is available upon request. (PDF 2094 kb)

## Abbreviations

BAG1: *Bcl2* associated athanogene 1
Bcl2: B-cell lymphoma 2
BEB: Bayes empirical bayes
bp: Base pairs
BP: Biological process
CAT: Catalase
CC: Cellular component
cds: Coding sequence
CEGMA: Core eukaryotic genes mapping approach
ELOVL6: Elongation of very long chain fatty acids protein 6
FDR: False discovery rate
GO: Gene ontology
Ig: Immunoglobulin
igNAR: Immunoglobulin new antigen receptor
LGMN: Legumain
MCL: Markov cluster algorithm
MF: Molecular function
MGAM2: Maltase-Glucoamylase (Alpha-Glucosidase)
Mya: Million years ago
ORF: Open reading frame
TIM22: Mitochondrial import inner membrane translocase subunit TIM22
TM6SF2: Transmembrane 6 superfamily member 2
