# Supplementary figures and images for: Comparative transcriptomics of elasmobranchs and teleosts highlight important processes in adaptive immunity and regional endothermy
Source: BMC Genomics. 2017 Jan 30;18:87. doi: 10.1186/s12864-016-3411-x (PMC5278576; doi:10.1186/s12864-016-3411-x)

3 **Fig S1b**

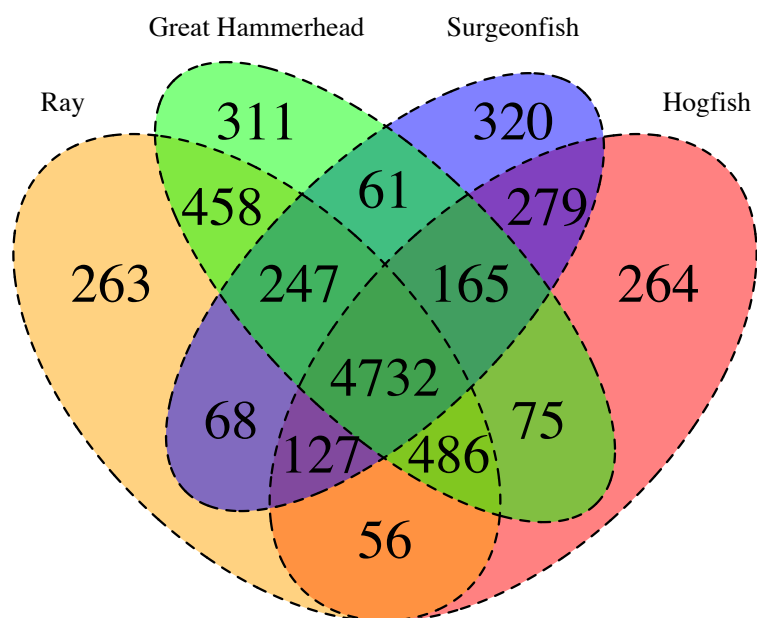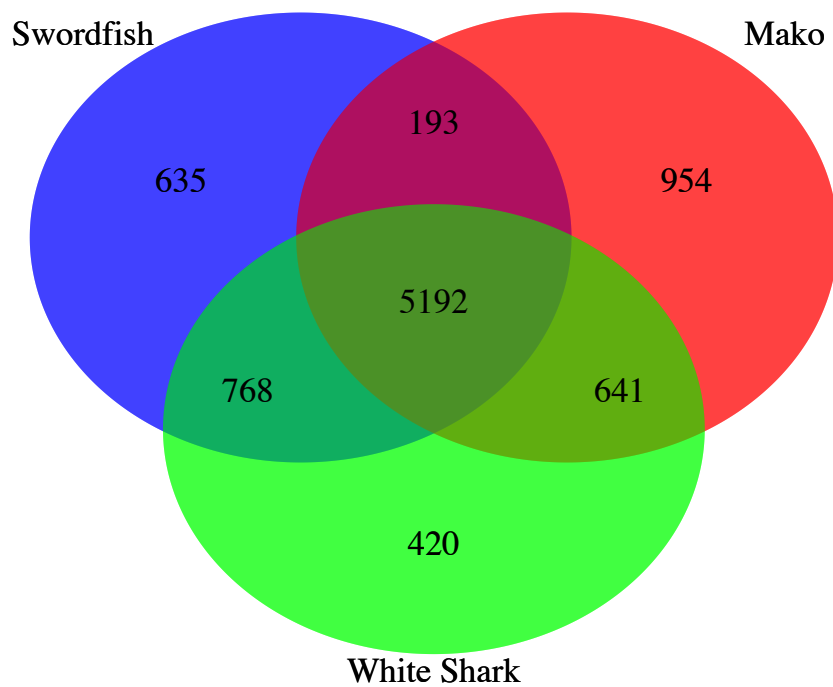

5

Supplement: Additional file 1: Figure S1a. — MCLBlastLINE sequence clusters shared among ectotherms. Figure S1b. MCLBlastLINE sequence clusters shared among endotherms. (PDF 573 kb) [file 12864_2016_3411_MOESM1_ESM.pdf]

Endotherms

Ectotherms

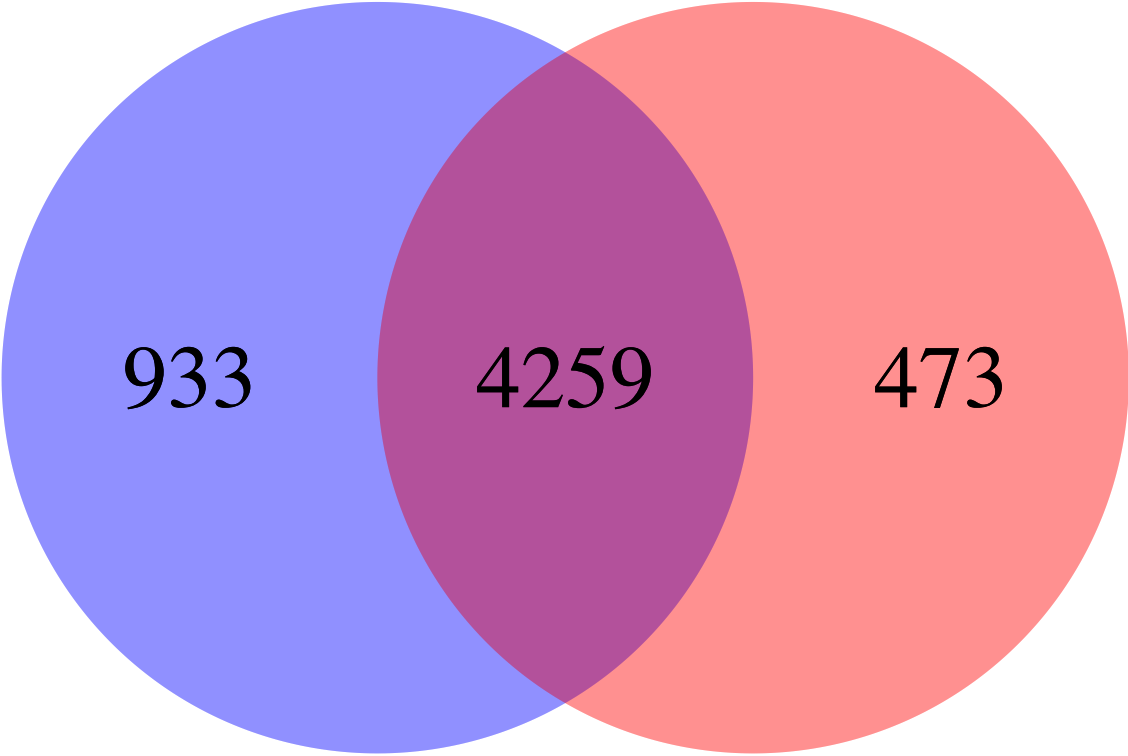

7

8

9

10

11

12

13

14

15

16

17

Supplement: Additional file 2: Figure S2. — MCLBlastLINE sequence clusters shared between endotherms (left) and ectotherms (right). (PDF 188 kb) [file 12864_2016_3411_MOESM2_ESM.pdf]

Fig. S3a.

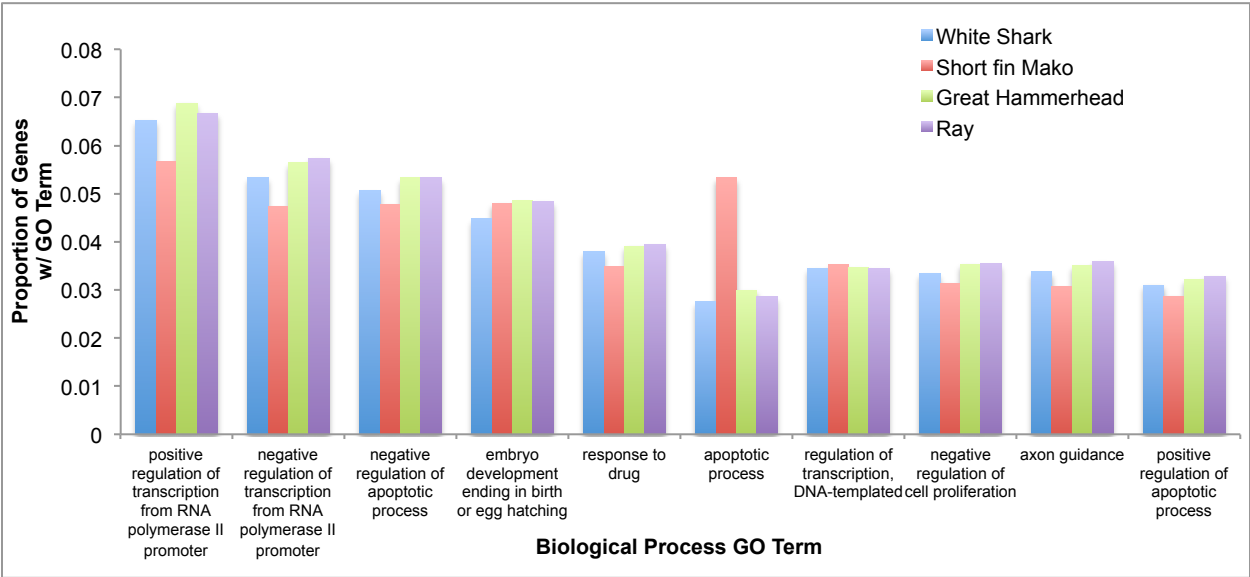

Fig. S3b.

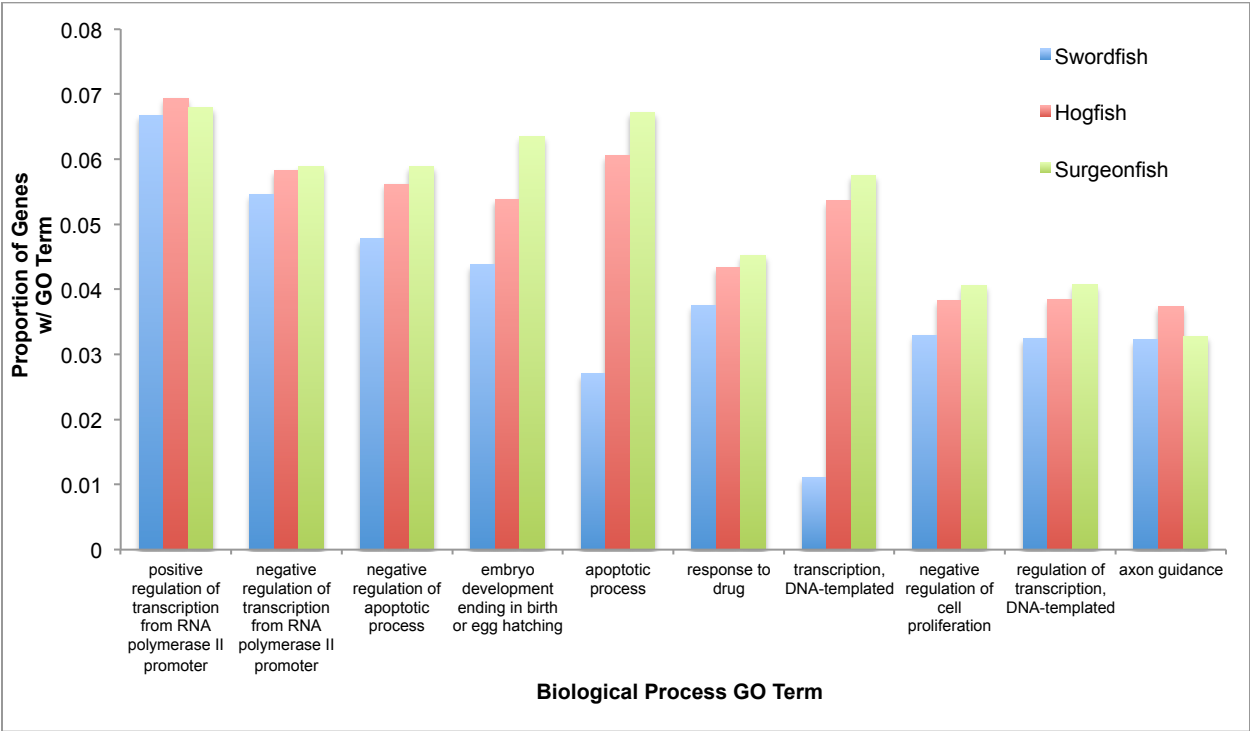

Supplement: Additional file 3: Figure S3a. — Top 10 Biological Process GO terms in elasmobranchs. Bars represent the proportion of contigs in each transcriptome that are annotated with the respective GO term. Figure S3b. Top 10 Biological Process GO terms in teleosts. Bars represent the proportion of contigs in each transcriptome that are annotated with the respective GO term. (PDF 311 kb) [file 12864_2016_3411_MOESM3_ESM.pdf]

Fig. S4a.

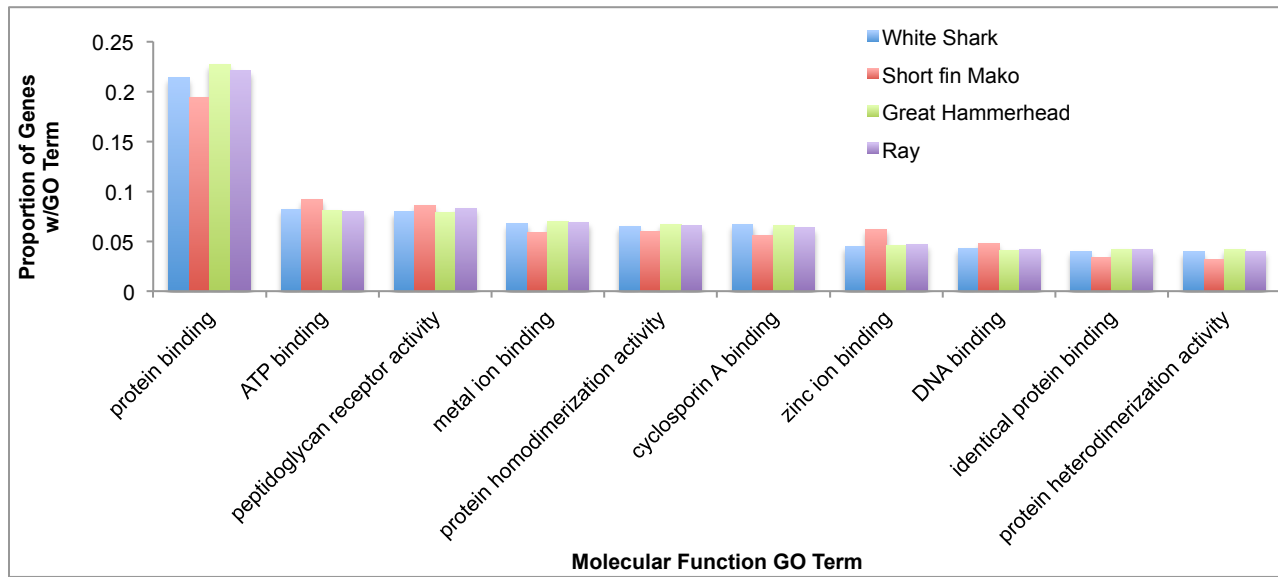

Fig. S4b.

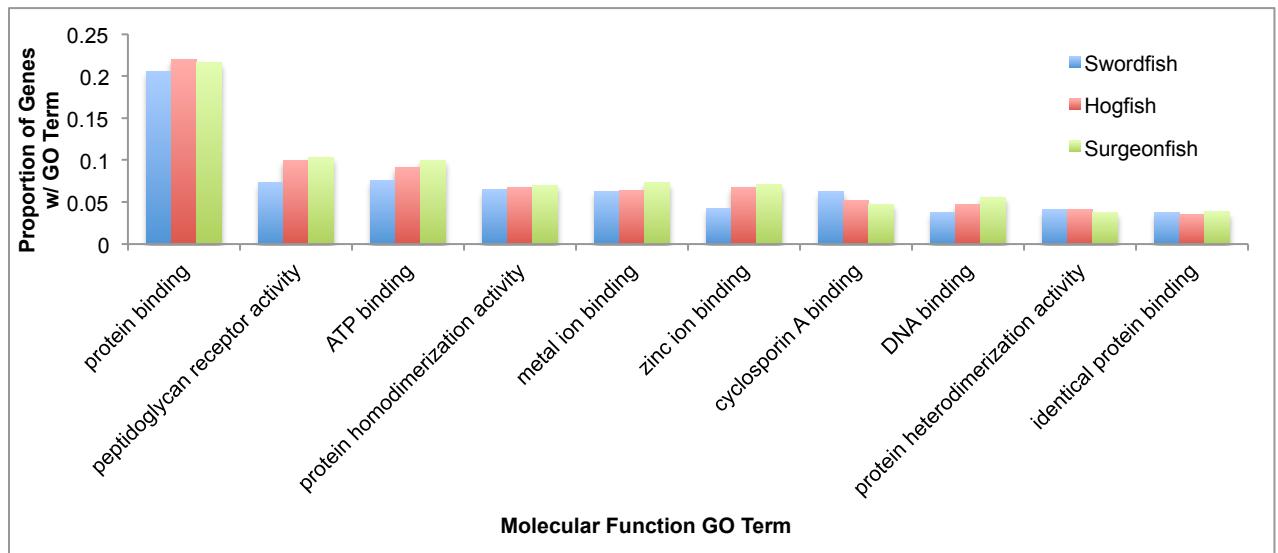

Supplement: Additional file 4: Figure S4a. — Top 10 Molecular Function GO terms in elasmobranchs. Bars represent the proportion of contigs in each transcriptome that are annotated with the respective GO term. Figure S4b. Top 10 Molecular Function GO terms in teleosts. Bars represent the proportion of contigs in each transcriptome that are annotated with the respective GO term. (PDF 409 kb) [file 12864_2016_3411_MOESM4_ESM.pdf]

Fig. S5a.

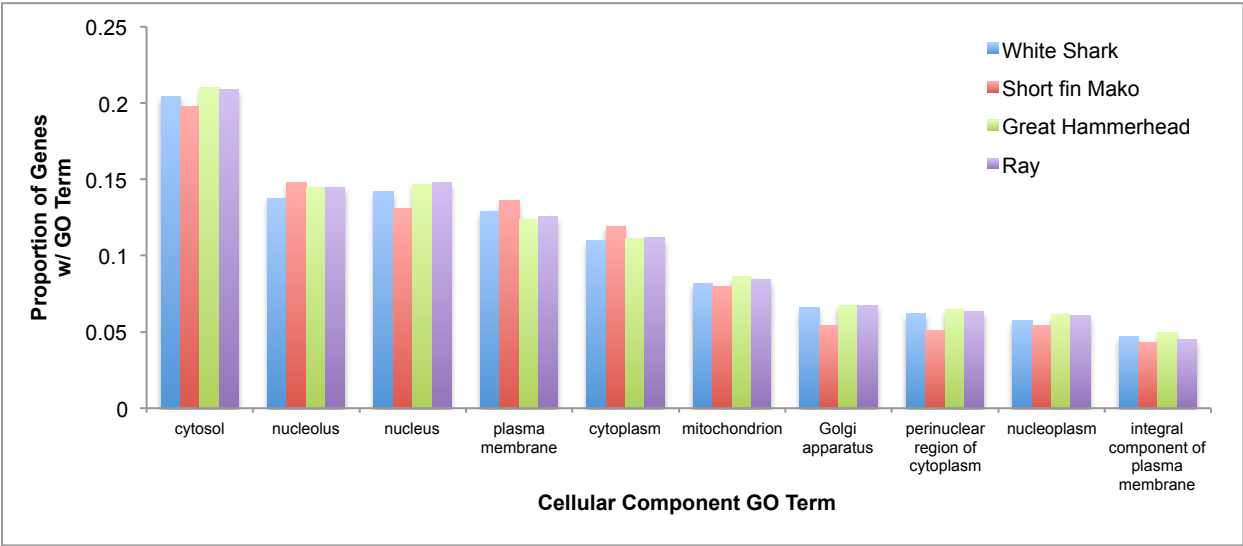

Fig. S5b.

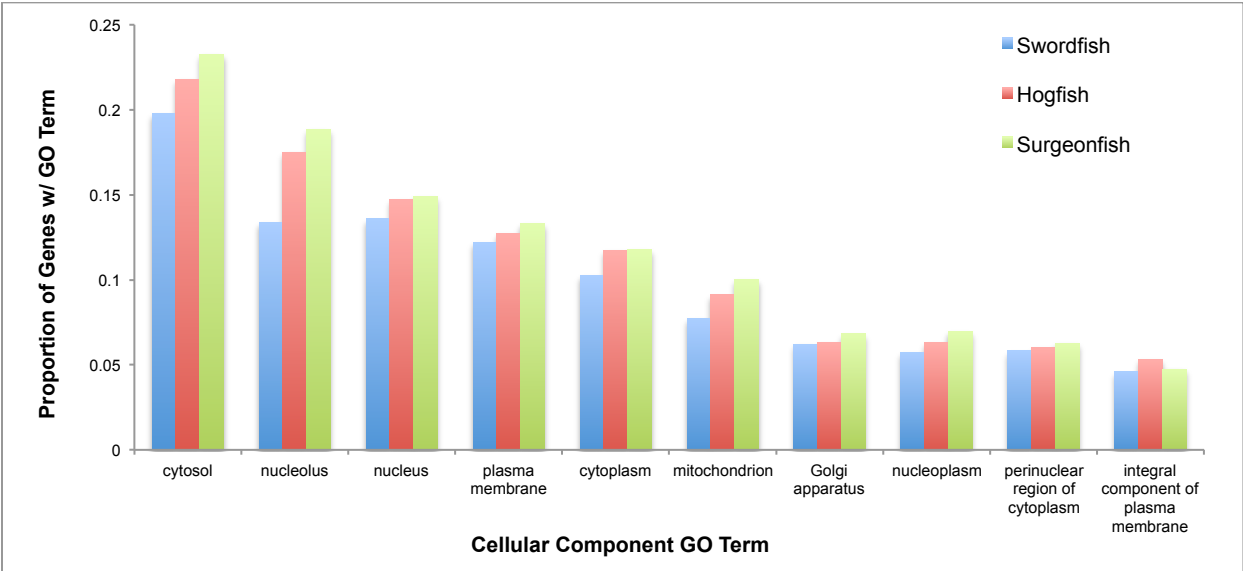

Supplement: Additional file 5: Figure S5a. — Top 10 most abundant Cellular Component GO terms for elasmobranchs. Histograms represent the proportion of contigs in each transcriptome that are annotated with the respective GO term. Figure S5b. Top 10 most abundant Cellular Component GO terms for teleosts. Histograms represent the proportion of contigs in each transcriptome that are annotated with the respective GO term. (PDF 290 kb) [file 12864_2016_3411_MOESM5_ESM.pdf]

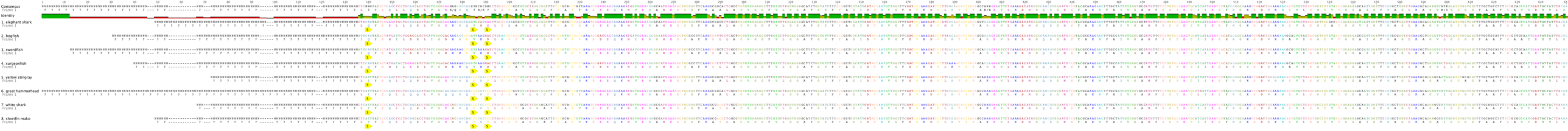

Supplement: Additional file 9: Figure S7. — Image of the alignment for mitochondrial import inner membrane translocase subunit TIM22 (Tim22), resulting from Probalign, as displayed in Geneious (v. 7.1.7). Bases that are located in gap regions or areas where the alignment is of low confidence are converted to N’s and then removed prior to running the branch sites test. The sites that are under selection according to BEB are flagged with yellow arrows at bases 139, 184, and 190 in the consensus sequence. The alignment file is available upon request. (PDF 1981 kb) [file 12864_2016_3411_MOESM9_ESM.pdf]

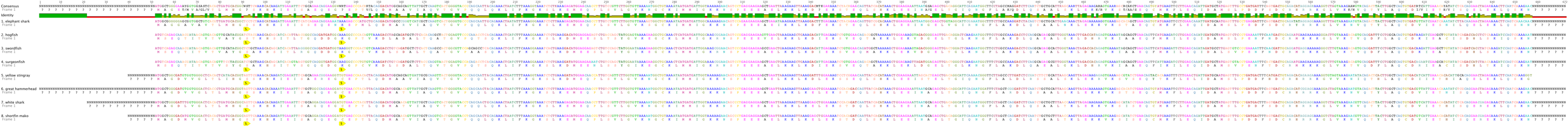

Supplement: Additional file 10: Figure S8. — Image of the alignment for BAG family molecular chaperone regulator 1 (Bag1), resulting from Probalign, as displayed in Geneious (v. 7.1.7). Bases that are located in gap regions or areas where the alignment is of low confidence are converted to N’s and then removed prior to running the branch sites test. The sites that are under selection according to BEB are flagged with yellow arrows at bases 91 and 133 in the consensus sequence. The alignment file is available upon request. (PDF 2094 kb) [file 12864_2016_3411_MOESM10_ESM.pdf]
