# Supplementary material for: Comparative transcriptomics of elasmobranchs and teleosts highlight important processes in adaptive immunity and regional endothermy
Source: BMC Genomics. 2017 Jan 30;18:87. doi: 10.1186/s12864-016-3411-x (PMC5278576; doi:10.1186/s12864-016-3411-x)
Supplement: Additional file 7: Table S6. — Genes with evidence of positive selection in elasmobranchs under the branch sites test after FDR filtering that remain significant at p < .10. Table S7. Genes with evidence of positive selection in endotherms prior to FDR filtering. (DOCX 19 kb) [file 12864_2016_3411_MOESM7_ESM.docx]

**Supplementary table S6: Genes with evidence of positive selection in elasmobranchs under the branch sites test after FDR filtering that remain significant at p<.10.**

| p_adj | p_value | dn/ds | Swissprot entry | Protein names | Gene names | Length aa | Length nt |
| --- | --- | --- | --- | --- | --- | --- | --- |
| 1.95E-02 | 2.89E-04 | 898.00 | Q99538 | Legumain (EC 3.4.22.34) (Asparaginyl endopeptidase) (Protease, cysteine 1) | LGMN PRSC1 | 433 | 1299 |
| 8.47E-02 | 1.79E-03 | ∞ | Q9BS26 | Endoplasmic reticulum resident protein 44 (ER protein 44) (ERp44) (Thioredoxin domain-containing protein 4) | ERP44 KIAA0573 TXNDC4 UNQ532/PRO1075 | 406 | 1218 |
| 4.84E-03 | 5.13E-05 | 810.51 | Q9JKW1 | Mitochondrial import inner membrane translocase subunit Tim22 | Timm22 Tim22 | 192 | 576 |
| 9.87E-02 | 2.51E-03 | 65.06 | Q92542 | Nicastrin | NCSTN KIAA0253 UNQ1874/PRO4317 | 709 | 2127 |
| 7.90E-02 | 1.34E-03 | ∞ | P08240 | Signal recognition particle receptor subunit alpha (SR-alpha) (Docking protein alpha) (DP-alpha) | SRPR | 638 | 1914 |
| 1.50E-02 | 1.90E-04 | 667.21 | Q99933 | BAG family molecular chaperone regulator 1 (BAG-1) (Bcl-2-associated athanogene 1) | BAG1 HAP | 345 | 1035 |
| 8.47E-02 | 1.98E-03 | ∞ | Q28852 | ATP synthase subunit g, mitochondrial (ATPase subunit g) | ATP5L | 103 | 309 |
| 8.47E-02 | 1.75E-03 | 462.29 | Q9Y3A2 | Probable U3 small nucleolar RNA-associated protein 11 (U3 snoRNA-associated protein 11) (UTP11-like protein) | UTP11L CGI-94 HDCMB12P | 253 | 759 |

Supplementary table S7: Genes with evidence of positive selection in endotherms prior to FDR filtering.

| p_adj | p_value | dn/ds | Swiss-Prot Entry | Protein names | Gene names |
| --- | --- | --- | --- | --- | --- |
| 1.00E+00 | 4.85E-02 | 19.19 | P55884 | Eukaryotic translation initiation factor 3 subunit B (eIF3b) (Eukaryotic translation initiation factor 3 subunit 9) (Prt1 homolog) (hPrt1) (eIF-3-eta) (eIF3 p110) (eIF3 p116) | EIF3B EIF3S9 |
| 2.11E-01 | 2.69E-03 | 9.93 | Q8BGA5 | KRR1 small subunit processome component homolog (HIV-1 Rev-binding protein 2 homolog) (KRR-R motif-containing protein 1) | Krr1 Hrb2 |
| 3.72E-01 | 5.51E-03 | 21.35 | Q569B7 | RWD domain-containing protein 4 | Rwdd4 Rwdd4a |
| 1.07E-01 | 9.10E-04 | 35.58 | Q9PWF7 | Catalase (EC 1.11.1.6) | cat |
| 2.11E-01 | 2.54E-03 | ∞ | Q9CR80 | Protein FAM32A (Ovarian tumor associated gene 12) (OTAG-12) | Fam32a Otag12 MNCb-3154 |
| 1.00E+00 | 4.43E-02 | 5.84 | Q5RBM1 | Dolichyl-diphosphooligosaccharide--protein glycosyltransferase subunit 2 (EC 2.4.99.18) (Dolichyl-diphosphooligosaccharide--protein glycosyltransferase 63 kDa subunit) (Ribophorin II) (RPN-II) (Ribophorin-2) | RPN2 |
| 7.32E-01 | 1.24E-02 | 2.41 | Q3ZBG7 | Signal recognition particle 19 kDa protein (SRP19) | SRP19 |
| 1.00E+00 | 3.15E-02 | ∞ | Q86UY6 | N-alpha-acetyltransferase 40 (EC 2.3.1.-) (N-acetyltransferase 11) (NatD catalytic subunit) | NAA40 NAT11 |
