# Supplementary material for: Comparative transcriptomics of elasmobranchs and teleosts highlight important processes in adaptive immunity and regional endothermy
Source: BMC Genomics. 2017 Jan 30;18:87. doi: 10.1186/s12864-016-3411-x (PMC5278576; doi:10.1186/s12864-016-3411-x)
Supplement: Additional file 8: Figure S6. — Image of the alignment for legumain (LGMN) resulting from Probalign, as displayed in Geneious (v. 7.1.7). Bases that are located in gap regions or areas where the alignment is of low confidence are converted to N’s and then removed prior to running the branch sites test. Note that the numbers for residues given in the manuscript correspond to positions in the human reference sequence; the sites under selection according to BEB are flagged with yellow arrows at bases 421 and 889 in the consensus sequence. The alignment file is available upon request. (PDF 4320 kb) [file 12864_2016_3411_MOESM8_ESM.pdf]

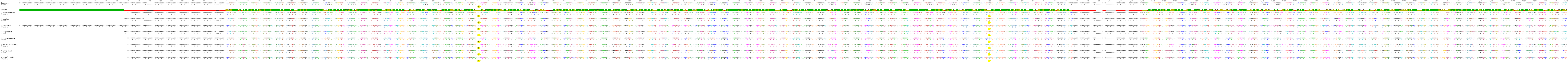
Consensus
Frame 1
Identity
1. elephant shark
Frame 1
2. hogfish
Frame 1
3. swordfish
Frame 1
4. surgeonfish
Frame 1
5. yellow stingray
Frame 1
6. great hammerhead
Frame 1
7. white shark
Frame 1
8. shortfin mako
Frame 1
